# LncRNA RP11-818O24.3 regulates proliferation and differentiation of hair follicle stem cells by targeting FGF2/PI3K/AKT pathway

**DOI:** 10.1371/journal.pone.0329647

**Published:** 2025-10-01

**Authors:** Linlin Bao, Haibo Zhao, Haiyue Ren, Chong Wang, Su Fang

**Affiliations:** 1 Department of Dermatology, Shenzhen People’s Hospital (The Second Clinical Medical College, Jinan University, The First Affiliated Hospital, Southern University of Science and Technology), Shenzhen, Guangdong, China; 2 Candidate Branch of National Clinical Research Center for Skin Diseases, Shenzhen, Guangdong, China; 3 Central South University, Changsha, Hunan, China; 4 Department of Gynecology, Shenzhen People’s Hospital (The Second Clinical Medical College, Jinan University; The First Affiliated Hospital, Southern University of Science and Technology), Shenzhen, Guangdong, China; Northwestern University, UNITED STATES OF AMERICA

## Abstract

Hair follicle stem cells (HFSCs) play critical roles in adult hair regeneration, owing to its self-renewal and multipotent differentiation properties. Emerging evidence has shown that long noncoding RNAs (LncRNAs) are implicated in biological processes such as proliferation, differentiation and apoptosis. However, the specific role of LncRNA RP11-818O24.3 in regulating HFSCs remains unclear. To explore the effect of LncRNA RP11-818O24.3 on HFSCs, stable LncRNA RP11-818O24.3 overexpression and knockdown HFSCs were established using a lentivirus vector system. The effect of LncRNA RP11-818O24.3 on proliferation was evaluated by Cell Counting Kit-8 (CCK8) and EdU incorporation experiments. The differentiation of HFSCs into neurons and keratinocyte stem cells was detected by immunofluorescence staining. We showed that LncRNA RP11-818O24.3 overexpression promoted the proliferation and inhibited cell apoptosis in HFSCs. High levels of LncRNA RP11-818O24.3 promoted the differentiation of HFSCs into CD34^+^K15^+^ keratinocyte progenitors and CD34^+^Nestin^+^neuron-specific enolase (NSE)^+^ neural stem cells. Additionally, LncRNA RP11-818O24.3 increased fibroblast growth factor 2 (FGF2) expression and the subsequent activation of the PI3K/AKT signaling pathway. These data demonstrated that LncRNA RP11-818O24.3 promotes self-renewal, differentiation, and the capability to inhibit apoptosis of HFSCs via FGF2 mediated PI3K/AKT signaling pathway, highlighting its potential role as a therapeutic strategy for treating hair loss diseases.

## Introduction

Hair loss (alopecia) is a common disorder that significantly affects the quality of life of humans [1]. Recent evidence suggests that the incidence of hair loss is gradually increasing and is onset at a younger age [2]. Common types of hair loss include androgen alopecia (AGA), female-pattern hair loss, alopecia areata and hair thinning [3]. It is reported that hair loss is a complex disease that can be triggered by both genetic and environmental factors [4]. Currently, effective treatment options available for hair loss are very limited. Therefore, it is worth exploring potential therapies to alleviate a growing societal burden.

The hair cycle constitutes three major phases: growth phase (anagen), regression phase (catagen), and resting phase (telogen) [5,6]. During the anagen phase, hair follicle stem cells proliferate and give rise to daughter cells that further differentiate into specific cell types such as keratinocytes, melanocytes, and inner and outer root sheath cells. This process is meticulously regulated by signaling pathways inclusive of Wnt/β-catenin [7], BMP, SHH, FGF, and TGF-β [8], as well as by key transcription factors such as SOX9. Of the cell types that form the hair follicle, hair follicle stem cells (HFSCs) play a critical role in the development of hair and the maintenance of the hair follicle cycle [9]. HFSCs are primarily located in the bulge region of the hair follicle, and senses hair growth signals that are secreted from dermal papilla cells during the telogen-to-anagen transition [10–12]. Additionally, HFSCs proliferate, and differentiate into various types of cells that organize the hair follicles, including keratinocyte progenitors and neural stem cells [13–16].

Long non-coding RNAs (LncRNAs) are a class of nucleotide RNA molecules (> 200) without protein-coding ability [17–19]. In recent years, LncRNAs have been reported to be implicated in various human diseases, including cancer, metabolic disorders and cardiovascular diseases [19,20]. Mechanistically, LncRNAs are able to orchestrate complex gene regulatory networks in cells in response to stimulation [21,22]. To this end, some progress has been made in the successful development of LncRNA-based pharmaceutical therapy [23]. For example, Yang and colleagues reported that LncRNA H19 could promote diabetic skin wound healing through inhibiting pyroptosis [24]. In addition, AC010789.1 boosts the biological function of HFSCs by inhibiting miR-21-5p and upregulating the Wnt/β-catenin signaling pathway [25]. Research by Lai *et al* also suggested that PlncRNA‑1 may promote proliferation and differentiation of HFSCs by upregulating TGF-β mediated Wnt/β-catenin signaling pathway [26]. These studies illustrate that LncRNAs hold promising therapeutical potential for hair loss via targeting HFSCs.

In the context of AGA, our genome-wide transcriptomic analysis identified LncRNA RP11-818O24.3 as one of the most significantly upregulated lncRNAs in AGA-affected scalp tissues compared to adjacent normal controls [27]. This dysregulation was further validated by qRT-PCR in a cohort of AGA patients. RP11-818O24.3 is genomically adjacent to the CRK and YES1 loci, which encode adaptor proteins and tyrosine kinases involved in growth factor receptor signaling (e.g., VEGF, TGF-β) and cytoskeletal dynamics-processes critical for HFSC activation and hair follicle regeneration.

In this study, we explored the potential function of LncRNA RP11-818O24.3 in HFSCs. We demonstrated that overexpression of LncRNA RP11-818O24.3 promotes proliferation and inhibits the apoptosis of HFSCs. Additionally, LncRNA RP11-818O24.3 induced differentiation of HFSCs into keratinocyte progenitors and neural stem cells. Mechanistically, we ascertained that RP11-818O24.3 exerts its effects via regulation of the fibroblast growth factor 2 (FGF2)-PI3K/AKT signaling pathway. These findings provide new insight into how RP11-818O24.3 modulates HFSC fate and illustrates its potential as a therapeutic target for hair loss diseases. The objective of this study is to elucidate the molecular mechanisms through which RP11-818O24.3 influences HFSC function in vitro.

## Materials and methods

### HFSCs culture and differentiation

HFSCs (HUM-iCell-s039) were purchased from iCell Bioscience Inc (Shanghai, China) and cultured in Primary Keratinocyte Culture System (PriMed-iCell-010, iCell). For differentiation induction, the HFSCs and LncRNA RP11-818O24.3 overexpression or knockdown HFSCs were switched to Defined Keratinocyte-SFM Growth Supplement medium (DK-SFM, 10785−012, Gibco) and DMEM/F12 medium (icell-0005-DZ, iCell) for 10 days. All cultures were maintained at 37°C in a humidified incubator containing 5% CO_2_. The medium was replaced every 2 days.

### Generation of stable LncRNA RP11-818O24.3 expressing HFSCs

Overexpression of LncRNA RP11-818O24.3 in HFSCs was performed using the lentiviral overexpression system. Briefly, LncRNA RP11-818O24.3 was cloned into the vector pLVX-IRES-puro. Lentivirus packaging and subsequent infection of HFSCs were performed as described previously [28,29]. The expression of LncRNA RP11-818O24.3 and FGF2 was detected using quantitative RT-PCR.

### Short hairpin RNAs for stable knockdown of LncRNA RP11-818O24.3 in HFSCs

To stably knock down endogenous LncRNA RP11-818O24.3 expression in HFSCs using a lentiviral system, a lentiviral vector for delivering short hairpin RNA (shRNA) targeting the LncRNA RP11-818O24.3 gene was constructed. Three small interfering RNA sequences targeting the LncRNA RP11-818O24.3 gene were designed, synthesized, and cloned into the pLVX-shRNA1 vector. Subsequently, lentiviral packaging and infection were performed as previously described [28,29]. The sequences of the shRNAs targeting LncRNA RP11-818O24.3 are shown in **Table 1**.

**Table 1:** shRNA target sequences.

Name	Sequence (5’-3’)
NC-F	GATCCCCTAGACTAGAATGTCCTATAGTCATTCAAGAGATGACTATAGGACATTCTAGTCTAGGTTTTTTG
NC-R	AATTCAAAAAACCTAGACTAGAATGTCCTATAGTCATCTCTTGAATGACTATAGGACATTCTAGTCTAGGG
shRNA1-F	GATCCCCTAGAGAATCAAGTGTTCCTATCATTCAAGAGATGATAGGAACACTTGATTCTCTAGGTTTTTTG
shRNA1-R	AATTCAAAAAACCTAGAGAATCAAGTGTTCCTATCATCTCTTGAATGATAGGAACACTTGATTCTCTAGGG
shRNA2-F	GATCCCCACCTCATTCAGTAAGCTCTACTTTTCAAGAGAAAGTAGAGCTTACTGAATGAGGTGGTTTTTTG
shRNA2-R	AATTCAAAAAACCACCTCATTCAGTAAGCTCTACTTTCTCTTGAAAAGTAGAGCTTACTGAATGAGGTGGG
shRNA3-F	GATCCTCAAAGCCGCTTGTTACCTGATCATTTCAAGAGAATGATCAGGTAACAAGCGGCTTTGATTTTTTG
shRNA3-R	AATTCAAAAAATCAAAGCCGCTTGTTACCTGATCATTCTCTTGAAATGATCAGGTAACAAGCGGCTTTGAG

### Cell transfection

FGF2 overexpression was performed by transfection of human FGF2 expression vector into targeted cells. Briefly, full-length human FGF2 cDNA fragment was ligated into PLVX-IRES-Puro vector. The transfections were performed using Lipofectamine 2000 Transfection Reagent (11668019, invitrogen) according to the manufacturer’s instructions. After 48 hours, the cells were used for subsequent experiments.

### Enzyme-Linked ImmunoSorbent Assay (ELISA)

FGF2, ChAT (choline acetyltransferase), and TH (tyrosine hydroxylase) protein levels were quantified using commercial human ELISA kits according to the manufacturers’ instructions. The following kits were used: human FGF2 ELISA kit (ELK1258, ELK Biotechnology, Wuhan, China), human ChAT ELISA kit (ELK4617, ELK Biotechnology, Wuhan, China), and human TH ELISA kit (ELK1974, ELK Biotechnology, Wuhan, China). Briefly, the stable cell lines with LncRNA RP11-818O24.3 knockdown and overexpression were cultured as described above [26]. The cell culture supernatant was harvested by centrifugation at the end of cultivation. Subsequently, the absorbance value at 450nm of each sample was obtained by microplate reader (DR-200Bs, Diatek, Wuxi, China) and the concentration of FGF2 was calculated based on the standard curve.

### Cell proliferation assay

Proliferation of HFSCs was evaluated using Cell Counting Kit-8 (CCK-8, C0038, Beyotime, Shanghai, China) and EdU incorporation experiment according to the manufacturer’s instructions. For the CCK-8 assay, transfected HFSCs were seeded in 96-well plates at a density of 1 × 10^4^ cells/well, and cultured for 48 hours. 10 μL of CCK-8 reagent was then added to each well and incubated for 2 hours. The absorbance values at 450 nm were obtained using a microplate reader (DR-200Bs, Diatek, Wuxi, China).

For the EdU incorporation experiment, transfected HFSCs were grown on glass slides and incubated with 50 μM EdU for 24 hours. After fixation, EdU was labeled using Click-iT (C10310-1, Ruibo, Guangzhou, China) reaction according to the manufacturer’s instructions, followed by Hoechst staining. The slides were then observed under a fluorescence microscope (IX51, Olympus, Tokyo, Japan) and analyzed using Image J software.

### Measurement of cell apoptosis

Apoptosis was detected using the Annexin V/7-AAD apoptosis kit (AO2001-09A-G, Tianjin Sanjian Biotechnology Co., Ltd., Tianjin, China) according to the manufacturer’s instruction. Briefly, harvested cell samples were washed with pre-chilled PBS (GNM20012, Genom, Hangzhou, China) and incubated with Annexin V and PI solution at room temperature for 10 minutes in darkness. The stained samples were then analyzed using CytoFLEX (Beckman).

### Immunofluorescence staining

For the cell differentiation assays, HFSCs were grown on glass slides for 48 hours. After fixation, the cells were permeabilized using 0.1% Triton X-100 (30188928, Sinopharm Chemical Reagent Co., Ltd., Shanghai, China) and sealed in 5% BSA solution (4240GR250, Biofroxx, Einhausen, Germany). The slides were then incubated with primary antibodies CD34 (AF5149, Affinity, 1:200), K15 (BF0225, Affinity, 1:100), nestin (Ab176561, Abcam, 1:200) and NSE (Ab79757, Abcam, 1:200) at 4°C overnight. On the subsequent day, the slides were treated with fluorescent secondary antibodies (Proteintech, Wuhan, China; ASPEN Biotechnology CO., LTD, Wuhan, China). After DAPI (D8417, Sigma, USA) staining, the cells were observed by fluorescence microscopy (IX51, Olympus, Tokyo, Japan) and analyzed using Image J software.

### Western-blot analysis

Western-blot assays were performed as described previously [30]. Briefly, cells were lysed in RIPA buffer (AS1004, ASPEN Biotechnology CO., LTD, Wuhan, China) containing phosphatase and proteinase inhibitors. Following this, the protein samples were separated on polyacrylamide gels and transferred onto PVDF membranes (IPVH00010, Millipore, MA, USA). The membranes were blocked with 5% milk in TBST for 1 hour and probed with primary antibodies FGF2 (DF6038, Affinity, 1:1000), AKT (#4691, CST, 1:5000), P-AKT (#4060, CST, 1:1000), P-PI3K (AB182651, Abcam, 1:500), Nestin(ab105389, abcam, 1:1000), CD34 (AF5149, affbiotech, 1:500), NSE (ab79757, abcam, 1:1000), K15 (BF0225, affbiotech, 1:1000) and GAPDH (ab181602, abcam, 1:10000) at 4°C overnight. Consequently, the membranes were incubated with corresponding HRP conjugated secondary antibody (AS1058, ASPEN Biotechnology CO., LTD, Wuhan, China), and the immunoreactive bands were visualized using ECL detection reagents (AS1059, ASPEN Biotechnology CO., LTD, Wuhan, China) as instructed. Protein expression levels were quantified using Image J software.

### RNA isolation and quantitative RT-PCR analysis

Total RNA was extracted from corresponding cell samples with TRIpure Total RNA Extraction Reagent (EP013, ELK Biotechnology, Wuhan, China) and cDNA was synthesized using EntiLink™ 1st Strand cDNA Synthesis SuperMix (EQ031, ELK Biotechnology, Wuhan, China) according to the manufacturer’s instruction. Subsequently, real-time PCR was performed using EnTurbo™ SYBR Green PCR SuperMix (EQ001, ELK Biotechnology, Wuhan, China) and a fluorescence quantitative PCR instrument (QuantStudio 6 Flex System, Life technologies, CA, USA). The PCR reaction was initiated at 94°C for 3 min followed by 40 cycles of denaturation at 95°C for 3 seconds and 60°C for 30 seconds. The sequences of qRT-PCR primers used are listed in Table 2. The GAPDH reference gene was used to normalize the gene expression data. The relative expression level for target genes was analyzed based on the 2^−ΔΔCT^ method as previously indicated [30].

**Table 2:** The sequences of human-specific qRT-PCR primers.

Name		Sequences
GAPDH	foward	5’-CATCATCCCTGCCTCTACTGG-3’
	reverse	5’- GTGGGTGTCGCTGTTGAAGTC-3’
K15	foward	5’-ATGCTGACCTGGAGGTGAAGA-3’
	reverse	5’-TCATACTTGAGCCTGAAGTCGTC-3’
CD34	foward	5’-GAAATCAAATGTTCAGGCATCAG-3’
	reverse	5’-CAGTAGACACTGAGGCCTCACC-3’
LGR5	foward	5’-TAGAAGTTTATCGGCATTGCAAG-3’
	reverse	5’-GGTATCGACCTGATATTGTTGCTAT-3’
LncRNA RP11-818O24.3	foward	5’-CCCCACCTCATTCAGTAAGCT-3’
	reverse	5’-GACCCCTCATTGGGTAATGAT-3’
FGF2	foward	5’-AAGAGCGACCCTCACATCAAG-3’
	reverse	5’-TTTCAGTGCCACATACCAACTG-3’

### Statistical analysis

All *in vitro* experiments were performed with at least three independent replicates. The data are presented as mean ± standard deviation (SD). GraphPad Prism (v8.0, GRAPHPAD SOFTWARE. LLC, Santiago, CA, USA) was used to conduct statistical analyses. The significance level was determined using either an independent student’s *t* test or ANOVA where appropriate. A calculated *p*-value < 0.05 was considered statistically significant.

## Results

### LncRNA RP11-818O24.3 supports HFSCs proliferation and inhibits apoptosis

**Fig 1:**
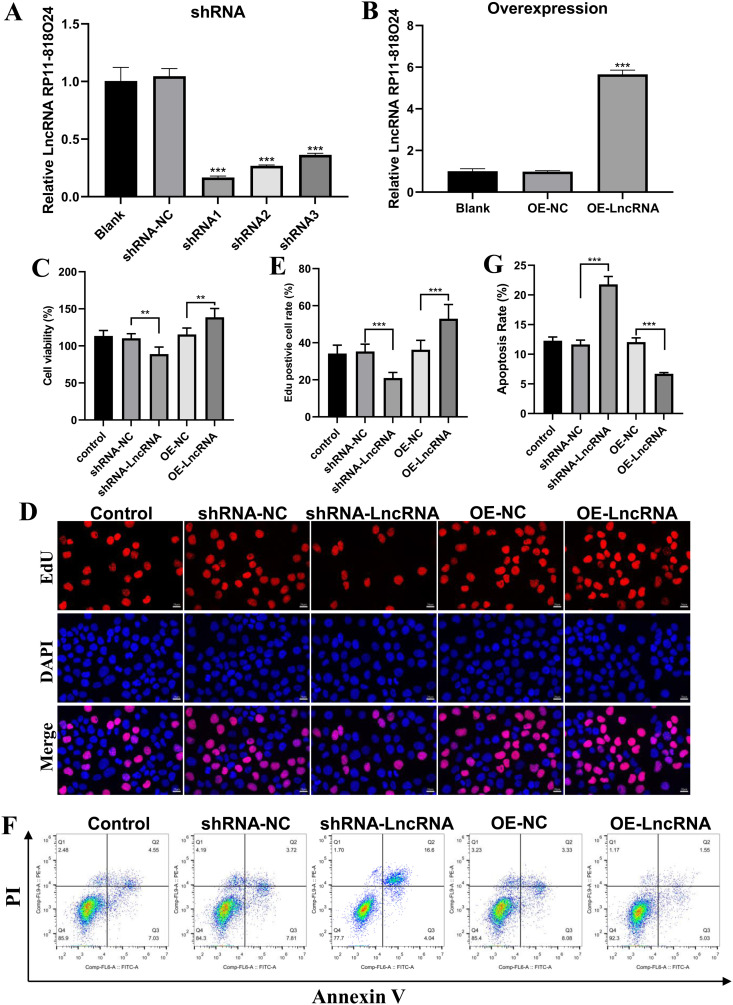
Effects of LncRNA RP11-818O24.3 on proliferation and apoptosis in HFSCs. **(A-B)** LncRNA RP11-818O24.3 overexpression and knockdown were conducted via lentiviral transfection. **(C)** CCK8 assay was used to detect the proliferation of hair follicle stem cells after LncRNA RP11-818O24.3 overexpression and knockdown. **(D)** The proliferation capacity of hair follicle stem cells was determined by EdU assay. **(E)** Statistical chart of EdU-positive cells evaluated using the ImageJ software. **(F)** Flow cytometry analysis of apoptosis using annexin V and propidium iodide staining following LncRNA RP11-818O24.3 overexpression and knockdown. **(G)** Quantitative analysis of cell apoptosis. HFSCs: hair follicle stem cells. Mean ± SD, **p < 0.01 and ***p < 0.001.

In order to characterize the effect of LncRNA RP11-818O24.3 in HFSCs, we first established stable HFSCs by knocking out or overexpressing LncRNA RP11-818O24.3 using a lentivirus-mediated system as described in the Methods. As shown in Fig 1A, the expression level of LncRNA RP11-818O24.3 significantly decreased in sh-LncRNA RP11-818O24.3 cells when compared with NC cells. LncRNA RP11-818O24.3 expression increased in overexpressed LncRNA RP11-818O24.3 (OE-LncRNA RP11-818O24.3) cells compared with NC cells (Fig 1B) ascertaining that the cell construction was successful.

Given the importance of LncRNA in cell proliferation, we next explored the impact of LncRNA RP11-818O24.3 on cell proliferation in HFSCs. The Cell Couting Kit-8 (CCK-8) assay result showed that LncRNA RP11-818O24.3 overexpression promoted HFSC proliferation, whereas the LncRNA RP11-818O24.3 knockdown inhibited cell proliferation (Fig 1C). In addition, EdU incorporation experiment further verified this finding (Fig 1D, E).

Next, we investigated how LncRNA RP11-818O24.3 affects cell apoptosis. Freshly transfected HFSCs were collected and stained with Annexin V/PI. Flow cytometry analysis showed that LncRNA RP11-818O24.3 knockdown resulted in a significantly increased apoptosis rate in HFSCs, whereas LncRNA RP11-818O24.3 directly protected HFSCs from cell death (Fig 1F, G). Collectively, these results show that LncRNA RP11-818O24.3 promotes HFSCs proliferation and inhibits apoptosis.

### LncRNA RP11-818O24.3 promotes HFSCs differentiation into keratinocyte progenitors

**Fig 2:**
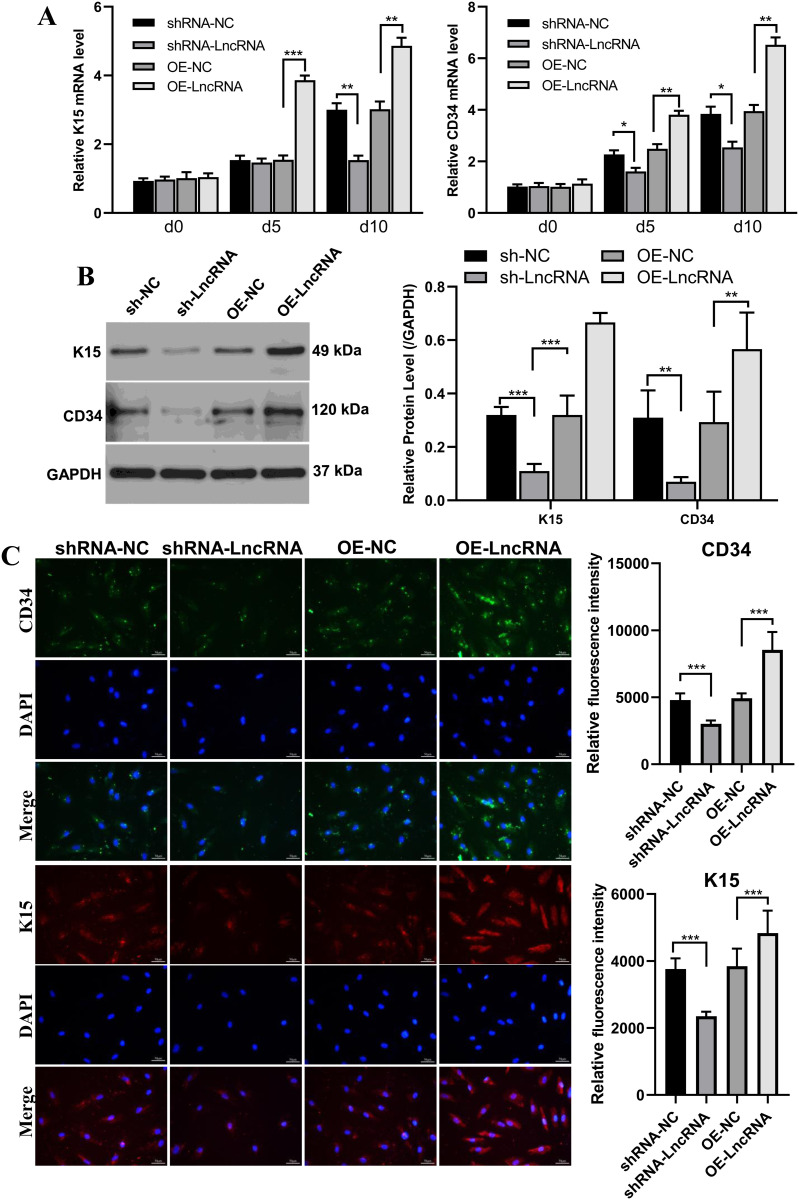
Effects of LncRNA RP11-818O24.3 on the expression of keratinocyte progenitor markers K15 and CD34. **(A)** Relative mRNA levels of K15 and CD34 were measured by qRT-PCR on days 0, 5, and 10 in cells with LncRNA knockdown (sh-LncRNA) or overexpression (OE-LncRNA), and their respective controls (shRNA-NC, OE-NC). **(B)** Protein expression levels of K15 and CD34 were assessed by Western blotting, with GAPDH as the loading control. Quantitative analysis of band intensity is shown on the right. **(C)** Representative immunofluorescence staining images of CD34 (green) and K15 (red) in different experimental groups, with DAPI (blue) for nuclear staining. Mean ± SD, *p < 0.05, **p < 0.01 and ***p < 0.001.

To assess the role of LncRNA RP11-818O24.3 in HFSC differentiation, the expression of keratinocyte progenitor markers K15 and CD34 at the mRNA and protein levels were ascertained. Quantitative RT-PCR revealed a time-dependent increase in K15 and CD34 transcripts with overexpression of LncRNA RP11-818O24.3, whereas knockdown of the lncRNA led to significantly reduced expression of both markers (Fig 2A). Western blot analysis also confirmed elevated protein levels of K15 and CD34 in the overexpression group and decreased levels with the knockdown (Fig 2B). Immunofluorescence staining further validated these findings at the cellular level, showing enhanced fluorescence intensity of K15 and CD34 in the OE-LncRNA group and reduced signal in the sh-LncRNA group when compared to controls (Fig 2C). Together, these data indicate that LncRNA RP11-818O24.3 promotes HFSC differentiation towards the keratinocyte progenitor lineage.

### LncRNA RP11-818O24.3 promotes HFSCs differentiation into neuron stem cells

**Fig 3:**
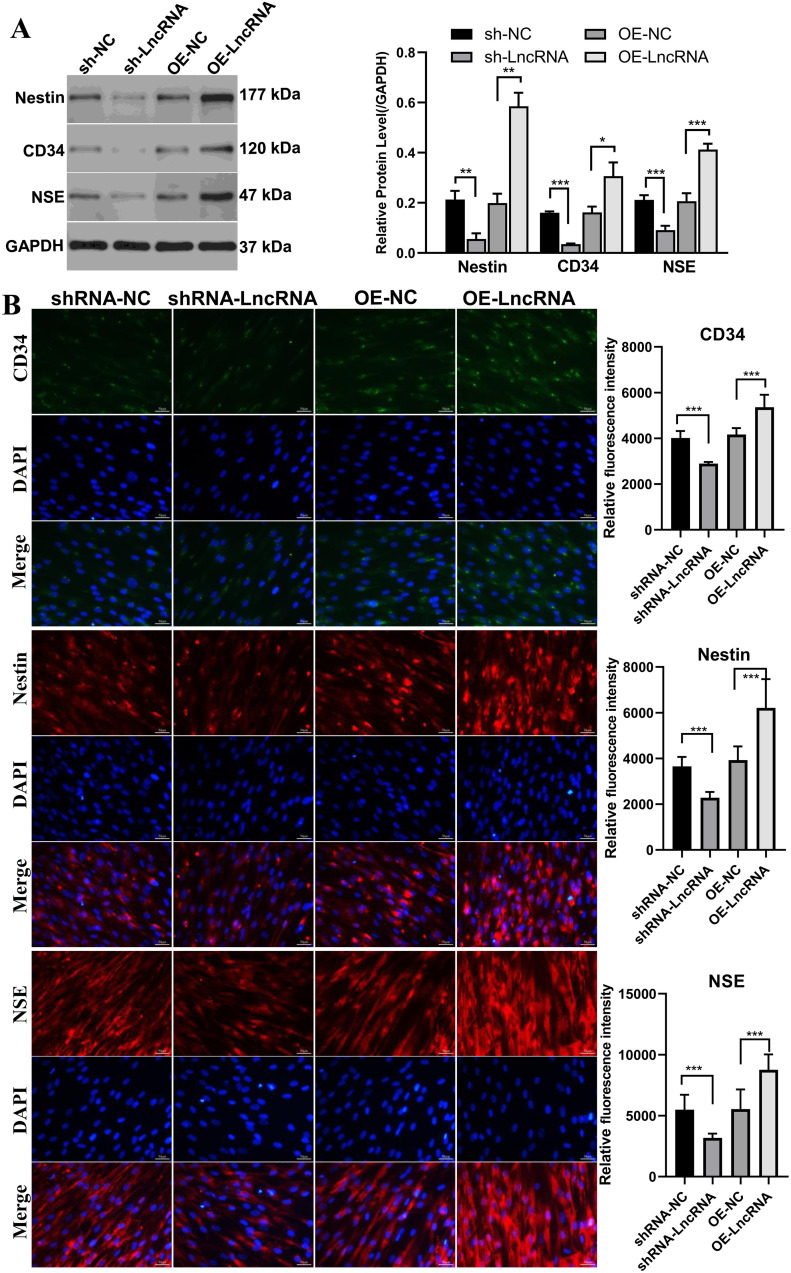
LncRNA RP11-818O24.3 promotes neural stem cell-like differentiation from hair follicle stem cells. **(A)** Western blot analysis of neural stem cell markers Nestin, CD34, and NSE in HFSCs following knockdown or overexpression of LncRNA RP11-818O24.3. **(B)** Representative immunofluorescence images showing CD34 (green), Nestin (red), and NSE (red) expression in HFSCs across the four groups. Nuclei were counterstained with DAPI (blue). Mean ± SD, *p < 0.05, **p < 0.01 and ***p < 0.001.

To investigate whether LncRNA RP11-818O24.3 regulates the neural differentiation potential of HFSCs, we assessed the expression of neural stem cell-associated markers following its knockdown or overexpression. Western blot and immunofluorescence analyses showed that overexpression of LncRNA RP11-818O24.3 markedly increased the levels of CD34, Nestin, and NSE, whereas the knockdown of LncRNA RP11-818O24.3 led to a significant reduction in their expression (Fig 3). These findings indicate that LncRNA RP11-818O24.3 facilitates the neural lineage commitment of HFSCs.

**Fig 4:**
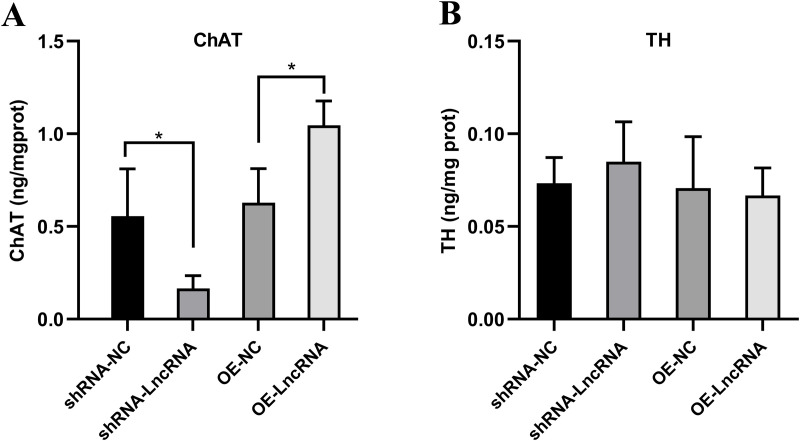
ELISA analysis of choline acetyltransferase and tyrosine hydroxylase protein levels in HFSCs following overexpression of LncRNA RP11-818O24.3. **(A)** Protein levels of ChAT. **(B)** Protein levels of TH. Mean ± SD, *p < 0.05, **p < 0.01 and ***p < 0.001.

To investigate whether RP11-818O24.3 modulates neuronal subtype specification, we quantified the secretion of ChAT and TH, markers of cholinergic and dopaminergic neurons, respectively, using ELISA. Overexpression of RP11-818O24.3 led to a significant increase in ChAT protein levels in the culture supernatant and TH levels remained unchanged (Fig 4), suggesting a potential role for RP11-818O24.3 in promoting cholinergic lineage specification.

### LncRNA RP11-818O24.3 upregulates FGF2 and PI3K/AKT in HFSCs

**Fig 5:**
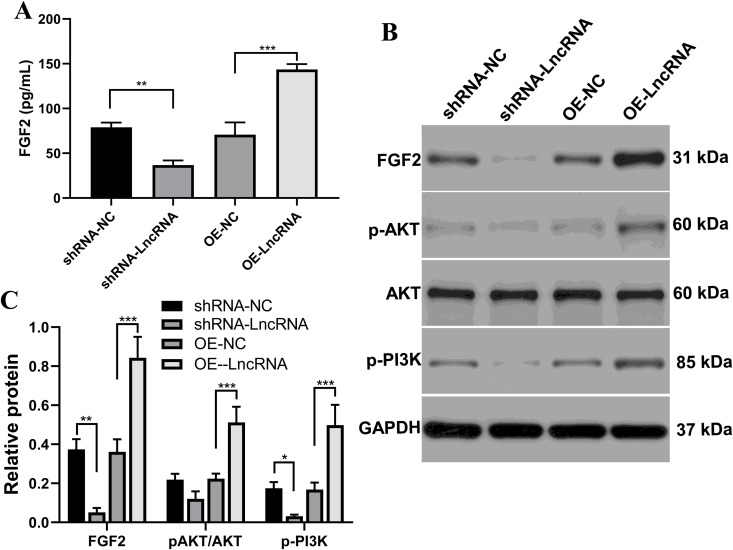
LncRNA RP11-818O24.3 upregulates FGF2 and PI3K/AKT in HFSCs. **(A)** Detection of FGF2 in cell culture supernatants by ELISA. **(B)** The protein expression of FGF2, P-PI3K, P-AKT and AKT was detected by western blot. **(C)** Statistical analysis of western blots. Mean ± SD, *p < 0.05, **p < 0.01 and ***p < 0.001.

Our previous *in vivo* studies showed that LncRNA RP11-818O24.3 promotes HFSC recovery via FGF2 and PI3K/AKT [31]. Here, we continued to investigate whether this signaling pathway was affected by LncRNA RP11-818O24.3 in HFSCs based on knockdown and overexpression analyses. FGF2 protein level in the cell culture supernatant detected by ELISA showed that levels in the LncRNA RP11-818O24.3 overexpression group was higher than the control group. Knockdown of LncRNA RP11-818O24.3 decreased the FGF2 concentration, suggesting that LncRNA RP11-818O24.3 triggers FGF2 expression and secretion (Fig 5A). Consistent with the above results, western-blot analysis also showed that FGF2 expression levels were closely related to LncRNA RP11-818O24.3. Additionally, compared with the control group, LncRNA RP11-818O24.3 overexpression induced a marked increase in the phosphorylation of both AKT and PI3K, whereas knockdown of LncRNA RP11-818O24.3 resulted in a decrease (Fig 5B, C). Collectively, these results indicate that LncRNA RP11-818O24.3 induced a significant upregulation of FGF2 and activation of PI3K/AKT signaling pathway in HFSCs.

### LncRNA RP11-818O24.3 mediates its effects via FGF2 in HFSCs

**Fig 6:**
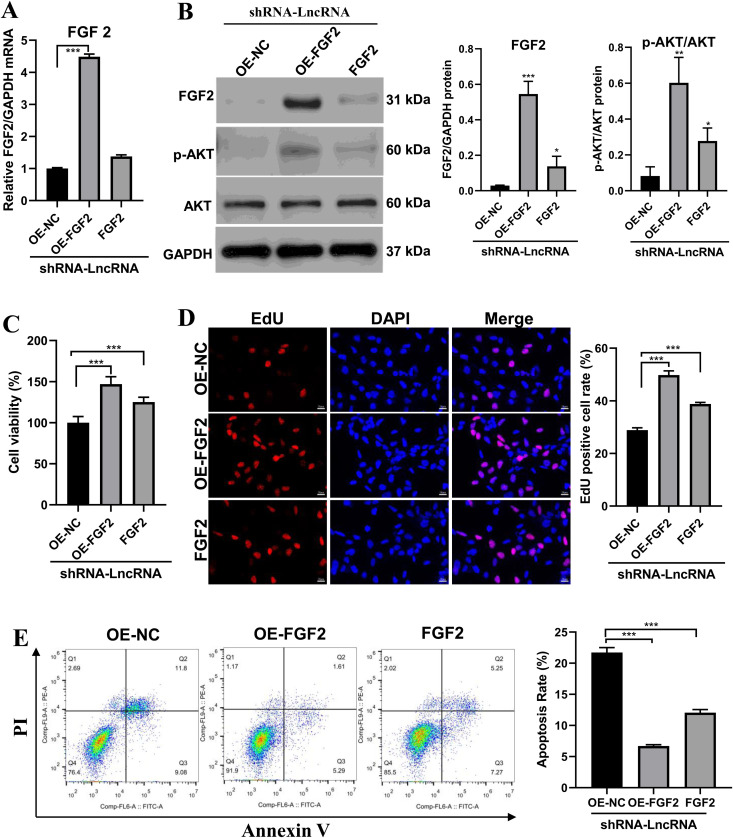
LncRNA RP11-818O24.3 exerts its regulatory effects via FGF2 in HFSCs. **(A)** Relative mRNA expression levels of FGF2 in HFSCs with an overespression of FGF2 based on the knockdown of LncRNA RP11-818O24.3. **(B)** The protein expression of FGF2, P-AKT and AKT was detected by western blot. **(C)** CCK-8 assay was performed to detect the cell proliferation in the indicated HFSCs. **(D)** Representative images and statistical chart of cell proliferation measured by EdU assay. **(E)** Representative images and statistical chart of cell apoptosis measured by flow cytometry. Mean ± SD, *p < 0.05, **p < 0.01 and ***p < 0.001.

To further characterize the role of FGF2 in the regulatory effect of LncRNA RP11-818O24.3 in HFSCs, an overexpression of FGF2 was generated in HFSCs based on the knockdown of LncRNA RP11-818O24.3. The identification results indicated that the FGF2-vector transfection was successful (Fig 6A-B). Notably, as compared with the control group, FGF2 (ELK1258, ELK Biotechnology) stimulation increased AKT phosphorylation, and the FGF2 overexpression group had the highest AKT phosphorylation level, suggesting that LncRNA RP11-818O24.3-mediated PI3K/AKT activation was related to the expression of FGF2. Subsequently, CCK-8 assay results showed that FGF2 promoted the proliferation of HFSCs (Fig 6C), which was further verified by EdU labeling experiment (Fig 6D). Cell apoptosis was improved by FGF2 administration or overexpression (Fig 6E). These results show that FGF2 promoted cell proliferation and inhibited apoptosis in HFSCs and these effects were closely related to their concentration.

**Fig 7:**
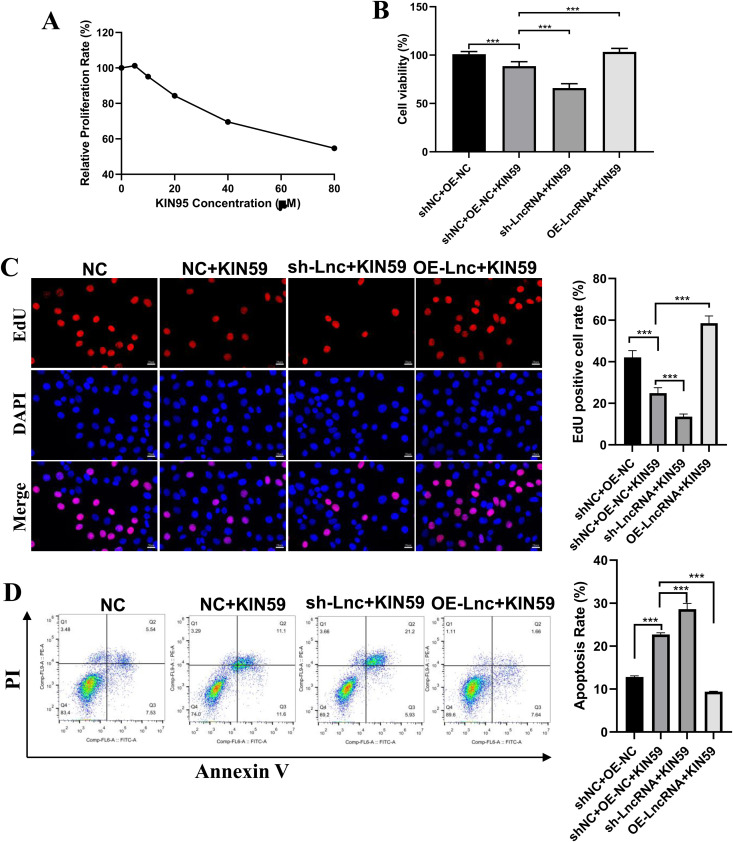
KIN95 treatment suppresses the phenotype caused by LncRNA RP11-818O24.3 overexpression in HFSCs. **(A)** CCK-8 results in HFSCs following KIN95 treatment. **(B)** CCK-8 results in the indicated HFSCs with or without KIN95 treatment. **(C)** Representative images and quantitative analysis of EdU assay in the indicated HFSCs with or without KIN95 treatment. **(D)** Representative images and statistical analysis of cell apoptosis in the indicated HFSCs with or without KIN95 treatment measured by flow cytometry. Mean ± SD, *p < 0.05, **p < 0.01 and ***p < 0.001.

We next performed rescued experiments using KIN95, an inhibitor of FGF2 signaling. KIN95 exhibited dose-dependent inhibition of cell proliferation (Fig 7A). In line with previous results, FGF2 overexpression cells had better cell viability (Fig 7B). However, this effect was significantly inhibited by KIN95 (Fig 7B). Additionally, KIN95 abolished the proliferative and anti-apoptotic effects mediated by FGF2 overexpression (Fig 7C-D).

These results indicate that LncRNA RP11-818O24.3 overexpression promotes cell proliferation and inhibits apoptosis through the FGF2-mediated PI3K-AKT signaling pathway.

## Discussion

In this study, we identify the long non-coding RNA RP11-818O24.3 as an unrecognized regulator of hair follicle stem cell biology and regeneration. Mechanistically, RP11-818O24.3 enhances HFSC proliferation, facilitates multilineage differentiation, and confers resistance to apoptosis. These findings uncover a critical role for RP11-818O24.3 in preserving HFSC function and orchestrating regenerative programs within the hair follicle niche.

Alopecia is an increasingly prevalent hair loss condition, and despite improved disease control, complete hair regrowth remains difficult to achieve. Owing to their potent self-renewal capacity and multilineage differentiation potential, the activation of endogenous hair follicle stem cells that reside within the bulge region of hair follicles has emerged as a promising regenerative strategy [32]. Disruption of HFSC homeostasis, manifested as impaired proliferation or heightened apoptosis, has been implicated in follicular degeneration and hair loss [32,33]. Therefore, therapeutic strategies focused on improving the survival, proliferation, and lineage commitment of hair follicle stem cells are crucial for the development of effective hair regeneration therapies.

Previous studies have identified the potential molecular pathways which may be associated with the proliferation of HFSCs inclusive of Wnt/β‑catenin, PI3K/AKT, c-Jun and TGF-β signaling pathways [34–36]. A growing body of evidence has also highlighted the regulatory roles of long non-coding RNAs in HFSC biology. For example, LncRNA5322, PlncRNA-1 and AC010789.1 have been shown to promote HFSC proliferation through MAPK1, PI3K/AKT and Wnt/β‑catenin signaling pathways respectively [25,26]. In this context, our study demonstrated that RP11-818O24.3 significantly enhances HFSC proliferation, as confirmed by EdU incorporation and CCK-8 assays. Moreover, RP11-818O24.3 overexpression conferred resistance to apoptosis, underscoring its importance in maintaining HFSC viability under stress conditions.

Mechanistically, LncRNA RP11-818O24.3 exerts its function via activation of PI3K/AKT signaling through upregulation of FGF2. Prior studies have established FGF2 as a key regulator of the PI3K/AKT axis, including in oncogenic contexts such as OS tumorigenesis, where the circ_001422/miR-195-5p/FGF2/PI3K/AKT pathway was implicated in promoting proliferation and metastasis [37,38]. In addition to PI3K/Akt signaling, FGF2 has also been shown to promote the differentiation of hair follicle stem cells into endothelial cells via activation of the STAT5 pathway [39], as well as maintaining the hair inductive capacity of dermal papilla cells by upregulating PDGFRα and cooperating with PDGF-AA to stimulate folliculogenic gene expression [40]. Our findings establish RP11-818O24.3 as a previously uncharacterized upstream regulator of FGF2, implicating it as a modulator of HFSC niche dynamics and hair follicle regeneration through coordinated control of PI3K/AKT signaling.

Beyond its role in promoting proliferation, RP11-818O24.3 also facilitates the multilineage differentiation of hair follicle stem cells. HFSCs possess the capacity to give rise to diverse cell types, including keratinocytes, neurons, epithelial cells, and smooth muscle cells [41]. Evidence from Guan *et al* suggests that lncRNA5322 induces HFSC differentiation into neuron cells through upregulating miR-21 [34]. PlncRNA-1 also increases HFSC differentiation by the Wnt/β-catenin signaling pathway [26]. In this study, our findings show that RP11-818O24.3 promotes the specification of HFSCs toward K15 ⁺ keratinocyte progenitors and nestin ⁺ /NSE⁺ neuronal progenitors, underscoring its broad regulatory influence on lineage commitment. These results are consistent with previous reports implicating long non-coding RNAs in directing HFSC fate through miRNA-mediated mechanisms and Wnt/β-catenin signaling pathways.

These findings suggest that RP11-818O24.3 may represent a promising therapeutic target for hair loss disorders by directly enhancing HFSC activation and lineage commitment. Its regulation of the FGF2–PI3K/AKT axis, a pathway with established druggability, further supports its translational potential. Unlike current treatments that act indirectly on the hair cycle, RP11-818O24.3 may complement existing therapies by promoting regeneration at the stem cell level, offering a mechanistically distinct and potentially synergistic approach to hair follicle restoration.

Nevertheless, this study has certain limitations. Our mechanistic investigations were conducted in vitro, and the in vivo relevance of RP11-818O24.3-FGF2-PI3K/AKT signaling in HFSC regulation still needs to be fully elucidated. While our data support a functional role for RP11-818O24.3, the comprehensive molecular mechanisms underlying its regulatory effects require further investigation. Future studies from our laboratory will focus on in vivo validation and detailed mechanistic dissection to advance our understanding of RP11-818O24.3 in HFSC biology and hair follicle regeneration.

In conclusion, this study demonstrated that LncRNA RP11-818O24.3 induces the proliferation and differentiation of HFSCs via the FGF2/PI3K/AKT signaling pathway, suggesting that a LncRNA RP11-818O24.3-based therapy could be influential in promoted HFSC mediated hair regeneration.

## Supporting information

S1 Fig Western blot raw images for Figs 5 and 6. (PDF)

## Abbreviations

AGA: androgen alopecia
HFSCs: hair follicle stem cells
LncRNAs: long non-coding RNAs
CCK-8: Cell Couting Kit-8
FGF2: fibroblast growth factor 2
ELISA: Enzyme-Linked ImmunoSorbent Assay
ChAT: choline acetyltransferase
TH: tyrosine hydroxylase
NSE: neuron-specific enolase
