# Supplementary figures and images for: LncRNA RP11-818O24.3 regulates proliferation and differentiation of hair follicle stem cells by targeting FGF2/PI3K/AKT pathway
Source: PLoS One. 2025 Oct 1;20(10):e0329647. doi: 10.1371/journal.pone.0329647 (PMC12488011; doi:10.1371/journal.pone.0329647)

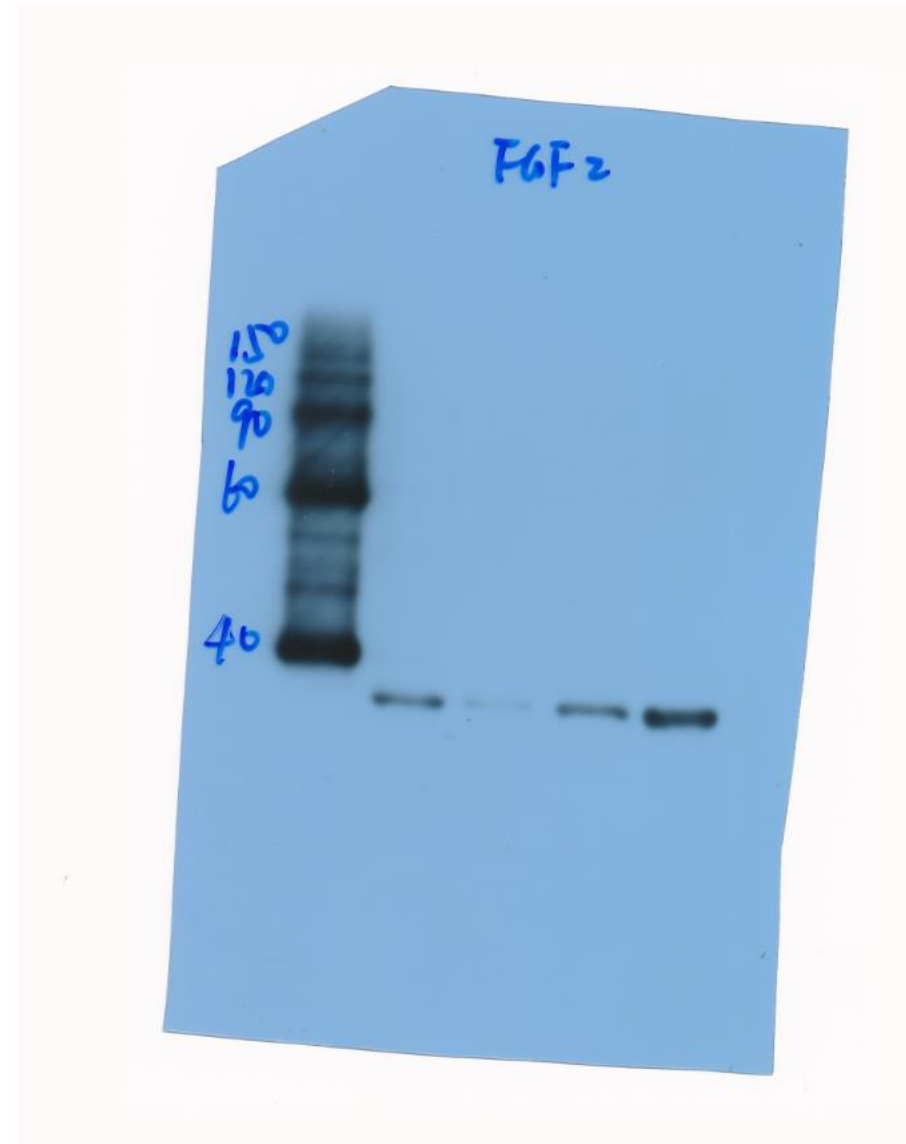

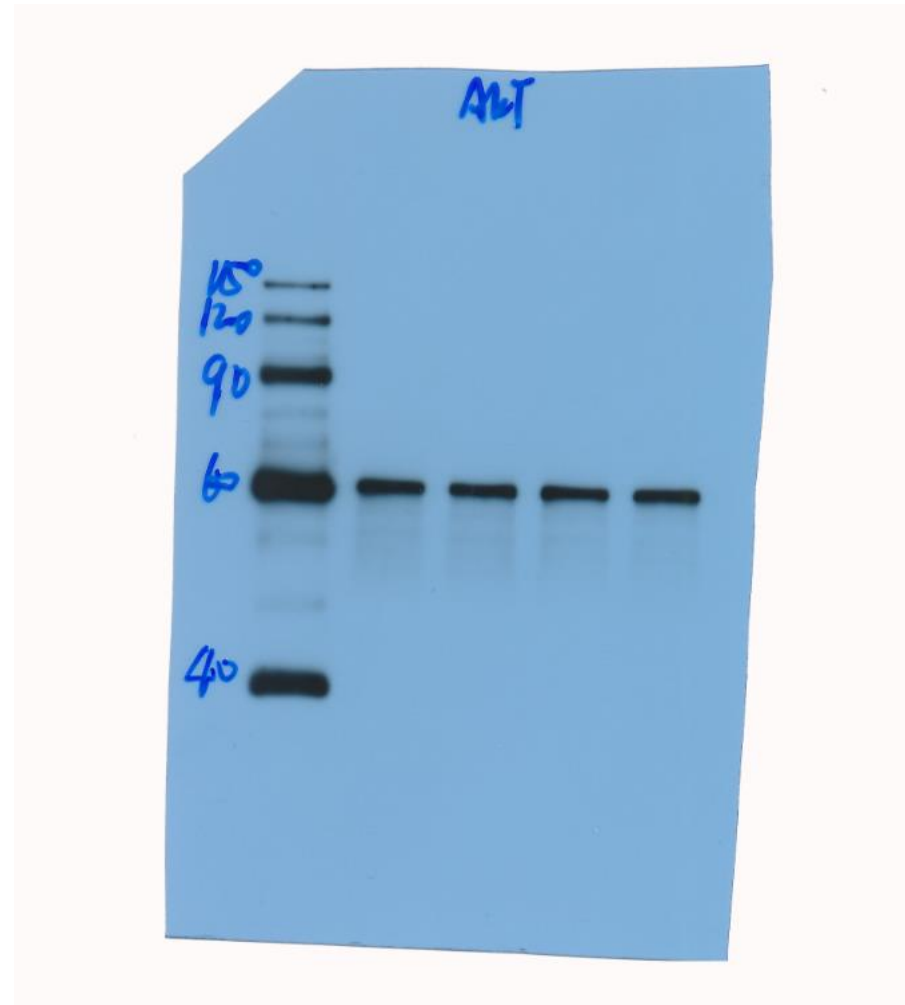

Figure 4

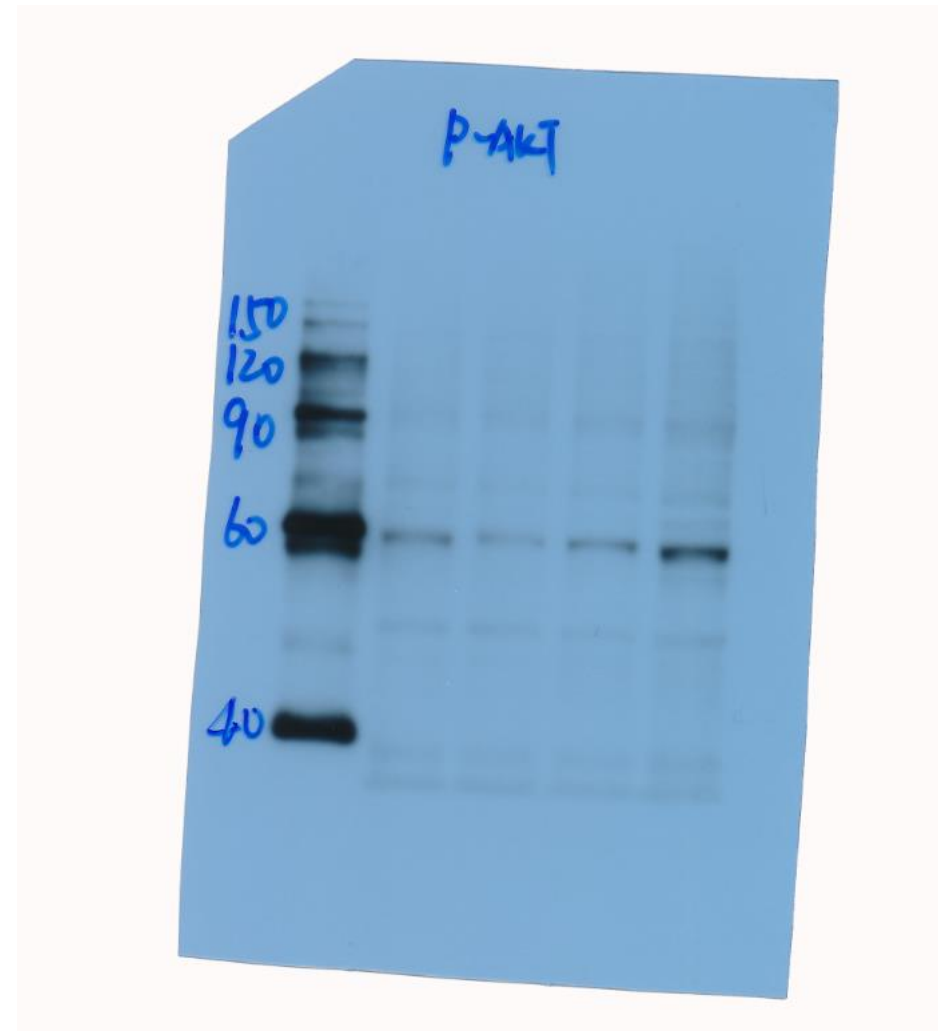

Figure 4

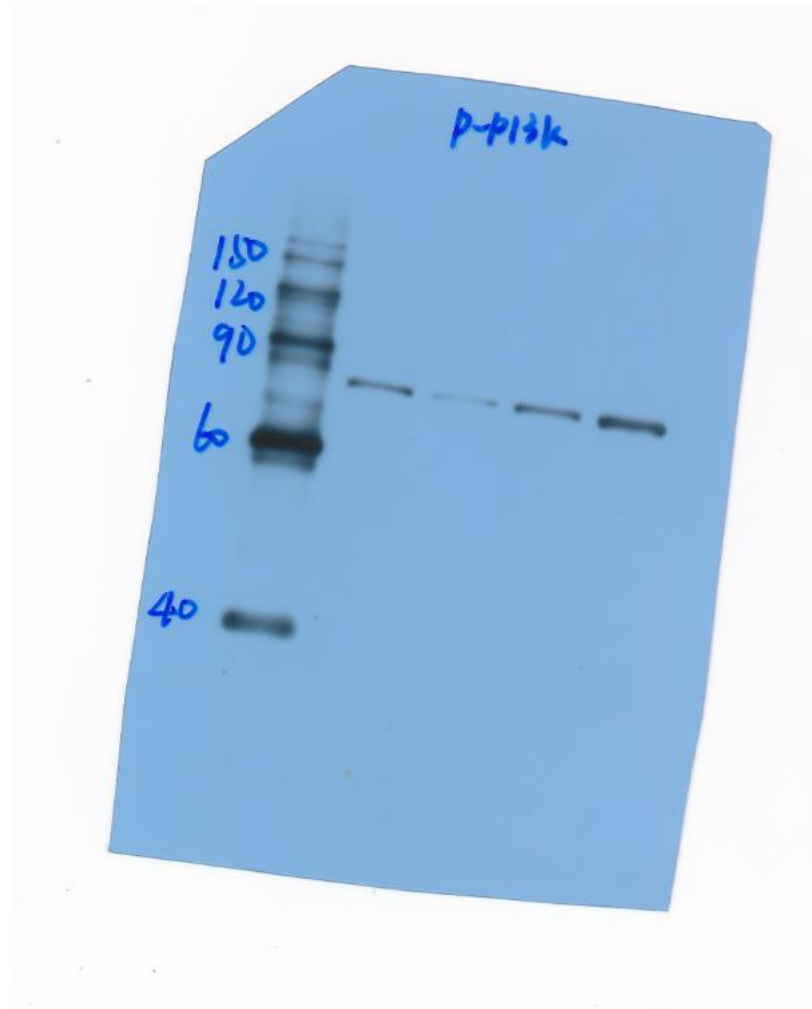

Figure 4

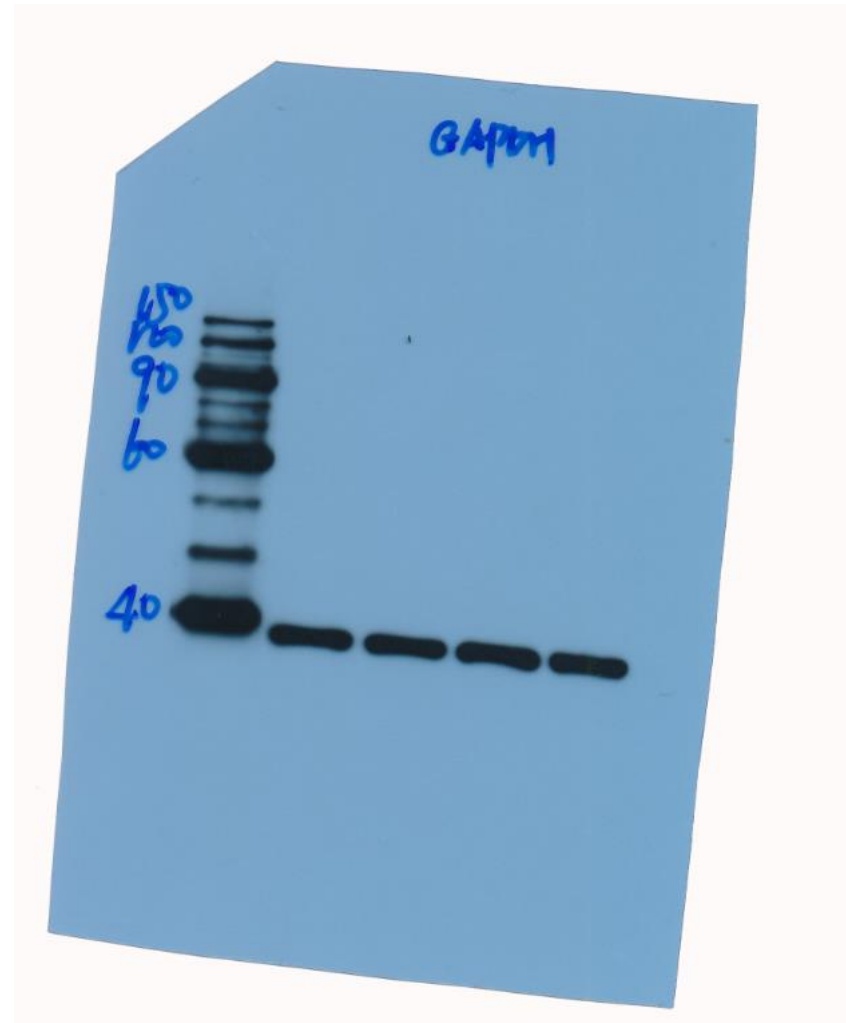

Figure 4

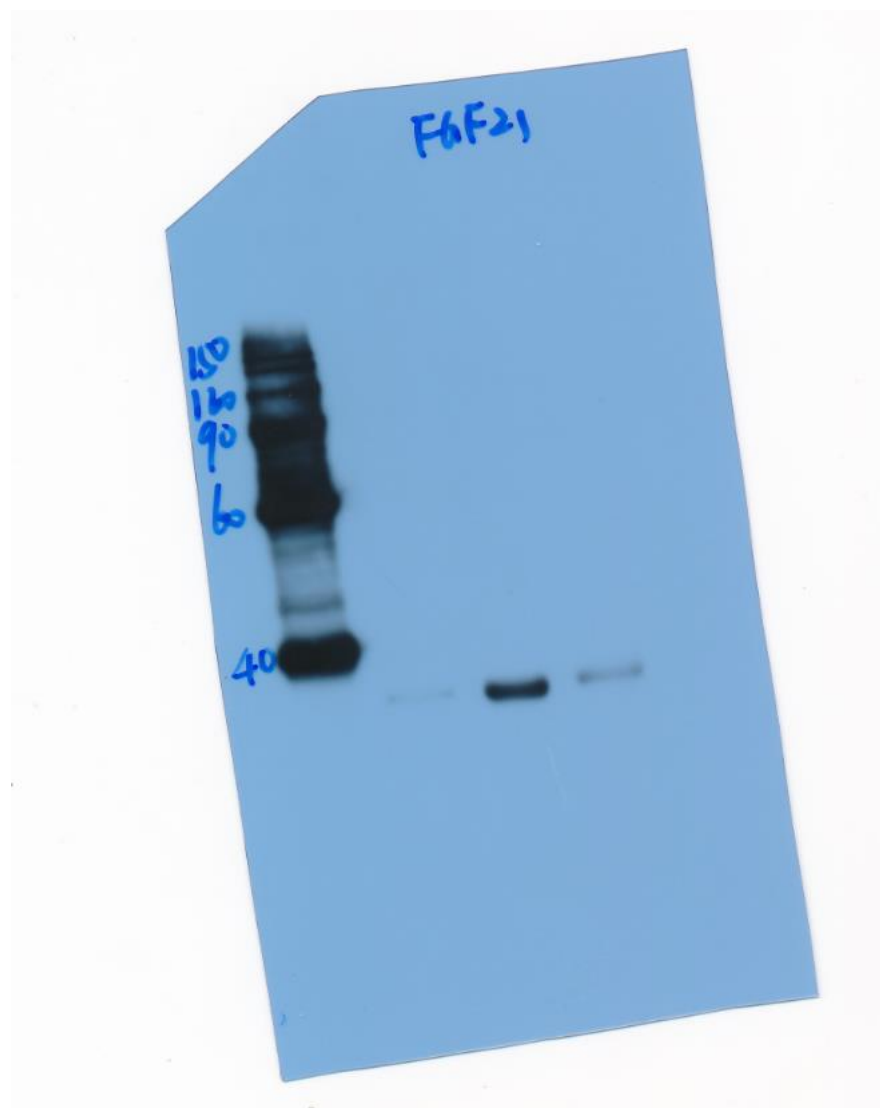

Figure 5

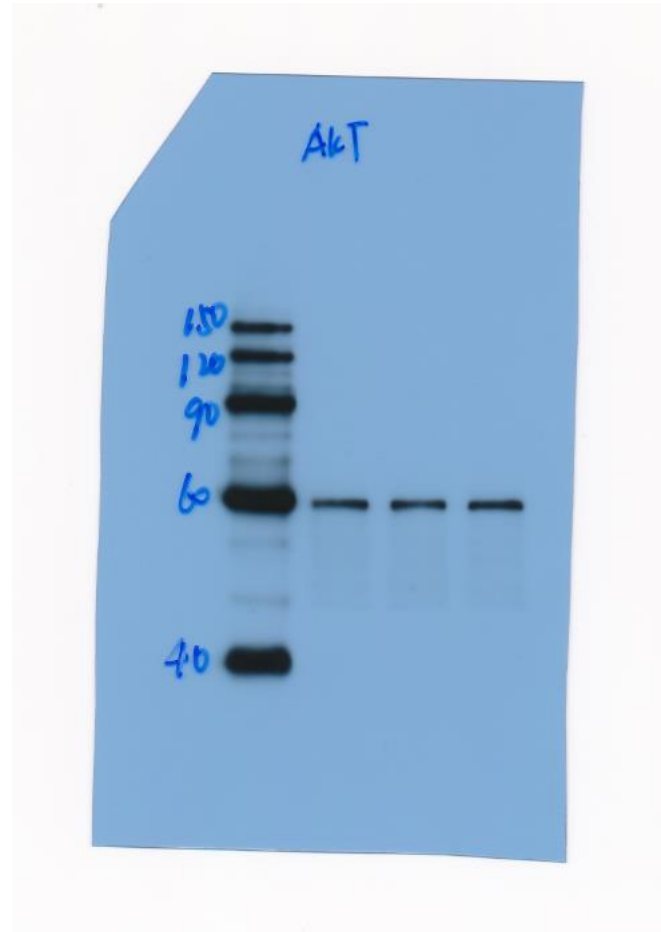

Figure 5

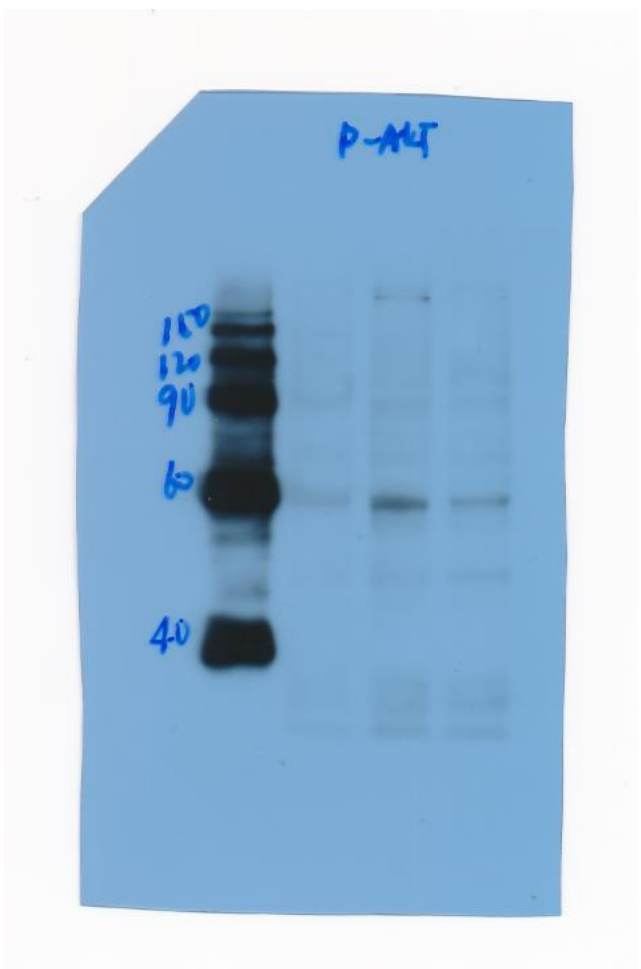

Figure 5

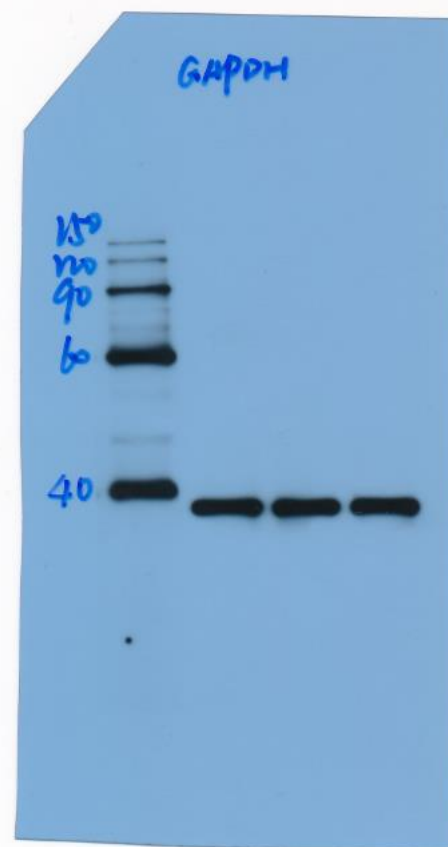

Supplement: S1 Fig — (PDF) [file pone.0329647.s001.pdf]
